# Dietary Spirulina (*Arthrospira platensis*) Modulates Survival, Growth, Reproductive Behavior, and Spawning Performance in Zebrafish, *Danio rerio*

**DOI:** 10.3390/ani16010098

**Published:** 2025-12-29

**Authors:** Ferdinando Flagiello, Maria Raggio, Marcello Diano, Serena Esposito, Maddalena Parente, Chiara Attanasio, Elena De Felice, Carla Lucini, Stefano Mazzoleni, Paolo de Girolamo, Livia D’Angelo, Antonio Palladino

**Affiliations:** 1 Department of Veterinary Medicine and Animal Production, University of Naples Federico II, 80137 Naples, Italy; ferdinando.flagiello@unina.it (F.F.); maria.raggio@unina.it (M.R.); chiara.attanasio@unina.it (C.A.); lucini@unina.it (C.L.); degirola@unina.it (P.d.G.); 2 M2M Engineering Sas, Business Innovation Center, Science Center, 80124 Naples, Italy; marcellodiano@m2mengineering.it (M.D.); serenaesposito@m2mengineering.it (S.E.);; 3 School of Biosciences and Veterinary Medicine, University of Camerino, 62032 Camerino, Italy; elena.defelice@unicam.it; 4 Department of Agricultural Sciences, University of Naples Federico II, 80055 Naples, Italy; 5 Department of Clinical Sciences and Translational Medicine, Faculty of Medicine and Surgery, University of Rome Tor Vergata, 00133 Rome, Italy

**Keywords:** aquaculture sustainability, functional feed, neuroendocrine modulation, reproductive physiology, teleost

## Abstract

In aquaculture and in laboratory settings, the development of sustainable and functional feeds is crucial in order to promote fish welfare, growth, and reproductive performance. Among natural dietary supplements, spirulina (*Arthrospira platensis*), a blue-green microalga rich in proteins, essential fatty acids, vitamins, and antioxidant molecules, represents a promising bioactive ingredient capable of influencing both physiological and behavioral traits. A 32-week longitudinal study was conducted on adult zebrafish (*Danio rerio*) to evaluate the effects of spirulina supplementation (5%, 25%, 50%, 75%, and 100%) compared to a standard diet. Parameters related to survival, growth, reproductive fitness, and reproductive behavior were assessed in vivo and supported by a morphometric analysis of the gonads. Supplementation with 5% improved survival rate and the Body Condition Index, while 25% supplementation increased survival, enhanced reproductive behavior and spawning success (140% egg production vs. control), and supported optimal gonadal development and gamete maturation timing. Higher percentages of spirulina (50–100%) seem to cause nutritional imbalance, impairing health and reproductive fitness. This study demonstrates that moderate spirulina supplementation (5–25%) supports health, reproductive physiology, and behavior in zebrafish in a dose-dependent manner. These results highlight the potential of spirulina as a functional supplement for precision nutrition approaches, with implications for fish welfare, reproductive performance, and aquaculture sustainability.

## Simple Summary

This research examined the impact of incrementally substituting a standard commercial diet with spirulina, a natural microalga renowned for its high protein and nutrient density, in zebrafish (*Danio rerio*). This longitudinal study aimed to assess how varying levels of spirulina integration in the diet affect fish health, growth, and reproductive success over a 32-week period. The findings revealed that while the partial substitution of commercial feed with spirulina maintained normal growth and reproduction, complete substitution resulted in poor health and decreased survival. Moreover, especially at lower substitution levels, spirulina has proven to have a beneficial effect on animal welfare and reproductive parameters. These results indicate that spirulina can be a beneficial supplement to fish diets, but the substitution levels need to be optimized to achieve the best outcomes, thereby enhancing sustainable feeding practices in aquaculture. This study underscores the potential introduction of natural ingredients like spirulina, in fostering animal welfare and also environmental sustainability in aquaculture settings.

## 1. Introduction

The advancement of functional feed development within aquaculture practices has garnered significant attention due to its potential to improve fish health, growth, and reproductive fitness. In this context, the incorporation of natural supplements into fish diets has emerged as a promising approach to enhance their overall performance and physiological well-being.

Spirulina (*Arthrospira platensis*), a blue-green microalga rich in proteins, vitamins, and essential nutrients, is widely recognized for its nutritional value and beneficial effects on aquatic species [1,2]. As a multicellular, filamentous cyanophyte, it grows rapidly even under conditions unfavorable to other microorganisms [3]. Its sustainable, cost-effective production and high nutritional quality have led to its increasing use as a dietary supplement for humans and animals. Spirulina contains 55–70% protein (dry weight) with a well-balanced essential amino acid profile, as well as significant amounts of polyunsaturated fatty acids (PUFAs), which are vital for growth and reproduction [4,5]. It is also rich in vitamins (B1, B2, B3, B6, B12, C, D, and E) [6] and bioactive pigments including carotenoids, chlorophylls, and phycocyanins, which exert strong antioxidant and immunomodulatory effects [7,8]. Furthermore, spirulina exhibits antibacterial and antifungal properties linked to its diverse bioactive compounds—PUFAs, polysaccharides, peptides, flavonoids, terpenes, alkaloids, and others—with the potential to contribute to reduced antibiotic use in aquaculture [9].

Reproductive behavior in fish represents a complex interplay of physiological status, hormonal regulation, and nutritional adequacy. Dietary composition has been shown to influence not only gamete quality but also behavioral components of reproduction, including courtship displays, mate selection, spawning readiness, and parental care. Essential fatty acids, particularly omega-3 and omega-6 polyunsaturated fatty acids (PUFAs), play crucial roles in neuroendocrine pathways that regulate reproductive behavior [10,11]. Adequate nutrition can enhance reproductive motivation, increase spawning synchrony, and improve mating success rates [12]. Therefore, functional feeds like spirulina may modulate reproductive behavior through both direct nutritional effects and indirect influences on hormonal profiles and energy availability [13]. Experimental studies in which yolk volume was manipulated demonstrated that yolk reserves are fundamental for survival and early larval growth [14]. Moreover, vitellogenin depletion, the main precursors of yolk proteins, impair embryonic development. This demonstrates that alterations in yolk reserve or composition affects critically larval development and viability [15].

In this study, we investigate the effect of the dietary integration, at different percentages, of spirulina in the standard diet of zebrafish (*Danio rerio*) on the survival, growth, health, and reproductive performance of zebrafish. Zebrafish are a well-established model organism in diverse research fields, including biomedicine, toxicology [16], and aquaculture [17]. Their suitability stems from the ease of maintaining large colonies under standardized, controlled conditions, minimizing confounding variables [18]. Under laboratory conditions, zebrafish live on average 3.5 years (up to ~5.5 years) and reach sexual maturity at 10–12 weeks post-fertilization, depending on diet, temperature, and husbandry. As batch spawners, they can reproduce multiple times throughout life; a two-week rematuration period between major spawning events is generally recommended to restore ovarian reserves. This short reproductive cycle and early maturity make zebrafish ideal for long-term reproductive and nutritional studies [19,20,21].

In this scenario, zebrafish represent a robust model for assessing the effects of spirulina supplementation and its relevance to aquaculture and fish conservation [22,23]. Spirulina has been tested in zebrafish and other fish species, as well as in various mammalian models [24,25,26]. The distinctive feature of our study is its longitudinal design that involved monitoring each tank over time relative to its initial condition. This approach enables a comprehensive evaluation of spirulina’s short- and long-term effects on survival, growth, and reproduction. Moreover, understanding how prolonged integration influences behavior provides valuable insights into sustainable aquaculture and conservation. Ultimately, the potential benefits of a spirulina-integrated diet may improve fish welfare, experimental reproducibility, and the sustainability of laboratory feeding protocols [27].

## 2. Materials and Methods

### 2.1. Animals

The experimental protocols involving animals were approved by the Italian Ministry of Health, protocol number [260/2024-PR]. Wild-type zebrafish (*Danio rerio*), strain AB, bred at the ZENOLAB facility of the Department of Veterinary Medicine and Animal Production at the University of Naples Federico II were used for this study. Animals were kept under standard conditions as described by Aleström [18]. Humane endpoints were defined in the project authorization (260/2024-PR) and consisted of the systematic evaluation of clinical signs, alterations in behavior, and the assessment of morphological parameters, including body condition score. Animals that met these criteria were humanely euthanized by administration of an overdose of methanesulfonate solution (MS-222, Tricaine-S^®^, Western Chemical Inc., Gujarat, India) at a concentration of 0.3 mg/L, dissolved in system water and buffered with sodium bicarbonate.

Death was confirmed by the complete cessation of opercular movement (gill breathing) for a minimum of ten minutes, followed by a secondary physical method (decapitation) to ensure irreversible death, in accordance with best practices for fish euthanasia.

### 2.2. Experimental Design

A total of 144 animals at sexual maturity (12 weeks of age) were divided into six groups. Each group included 12 males and 12 females housed in separate tanks, which were morphologically identified according to Avdesh et al. [28].

The food SDS Diets 400, a commercial formulation commonly used in aquatics research, possesses the nutritional composition, energy content, minerals, and vitamins that are listed in Table 1 (zebrafish basic feeding/husbandry information is available at https://www.akronom.hu/_data/1478525588.pdf) (accessed on 20 December 2025). *Arthrospira platensis* product, in micronized powder form, was provided by M2M Engineering (Naples, Italy) and the main composition is showed in Table 2. For convenience, we refer to spirulina over the text instead of *A. platensis*.

Fish were fed an amount of feed equal to 5% of their body weight per day, split into twice a day (2.5% morning + 2.5% afternoon), for an experimental period of 32 weeks. The six experimental diets were formulated by progressively replacing the standard feed SDS Diets 400 with micronized *Arthrospira platensis* (spirulina) powder on a mass-for-mass substitution approach. The resulting treatments were as follows:

S0, control group: 100% SDS Diets 400;
S100: 100% *Arthrospira platensis* (spirulina);
S75: 25% SDS + 75% spirulina;
S50: 50% SDS + 50% spirulina;
S25: 75% SDS+ 25% spirulina;
S5: 95% SDS + 5% spirulina.

The ingredient mixtures were prepared by thoroughly homogenizing the commercial feed with spirulina powder, followed by pelleting and drying at low temperature. All diets were stored at 4 °C until use. The composition of each diet was calculated as the weighted mean of each ingredient of the two diets as provided in Supplementary Table S1. The feed–spirulina mixture was administered in water to the fish of the different treatment groups. All tanks housing the experimental animals were siphoned every evening, with a water renewal of approximately 50% of the total volume. The replaced water originated from a recirculating system in which pH, temperature, and conductivity were continuously monitored and maintained within the standard ranges recommended for zebrafish husbandry. The same water renewal procedure was applied to the control tanks to avoid any potential confounding effects among experimental groups. In addition, the levels of ammonium (NH_4_^+^), nitrates, and nitrites were tested weekly in each tank prior to water replacement. In no case did the measured values exceed the limits recommended by standard zebrafish husbandry protocols [18,23].

### 2.3. Survival Rate

For each experimental group, we evaluated the survival rate by means of Kaplan–Meier method. Briefly, based on daily observations, we recorded the dead animals in a specific group. We arbitrarily divided the experimental period into 14 observation time points, corresponding to the bi-weekly weight recording intervals. At the end of each two weeks interval, the number of dead animals was recorded. Finally, to assess the survival rate of a group, the following formula was applied:

(1) $$Survival\;rate=\;\frac{Number\;of\;living\;individuals}{Total\;number\;of\;individuals}*100$$

### 2.4. Reproductive Behavior and Fitness Assessment

To assess reproductive behavior and spawning performance, every 2 weeks (7 days after weight and length recording), animals were paired for mating trials. Twelve hours before mating, animals were placed in breeding tanks with two males and two females separated by a transparent divider. To maintain the light–dark cycle and minimize stress, tanks were covered with a dark cloth overnight. The following morning, both the dark cloth and the divider were removed to allow behavioral interactions. Spawning readiness and activity were qualitatively assessed during the 3 h observation period, which involved monitoring the presence or absence of active mating behavior and egg deposition. An observation period of three hours, from the removal of the separator to egg collection, was maintained to ensure the proper sequence of mating behavior and to allow optimal egg deposition and fertilization in zebrafish [23]. The released eggs were carefully collected in a Petri dish and immersed in Methylene blue diluted in water system (0.01%). The eggs were observed under a stereomicroscope and counting was carried out for each experimental group. The reproductive performance parameters were measured as follows:

- Percentage of total spawned eggs: Relative egg production compared to S0 group over the entire experimental period;
- Average number of spawned eggs per mating;
- Percentage of fertilized eggs per mating: Proportion of successfully fertilized eggs among total spawned eggs, assessed by visual inspection 24 h post fertilization (presence of cell division and normal cleavage);
- Average number of hatched larvae per mating: mean number of viable larvae that successfully hatched (typically 48–72 h post fertilization).

### 2.5. Body Measurements

Every two weeks from the start of the experimental trial, all animals were anesthetized using a solution of methanesulfonate (MS-222, Tricaine-S^®^, Western Chemical Inc., Gujarat, India) at a concentration of 0.1 mg/L, dissolved in system water and buffered with sodium bicarbonate. Once the animals showed no response to the tail fin pinch reflex and lost balance, they were quickly transferred to a laboratory scale for weighing. Additionally, their length was measured using a digital caliper. At each time point (*n* = 14), the length and weight of each animal were recorded.

Weight and total length data were used to determine the Body Condition Index (BCI) by applying the following formula [29]:

(2) $$BCI:\;\frac{Wet\;Body\;Weight\;\left(mg\right)}{Total\;body\;lenght\;\left(mm^{3}\right)}\cdot \;100$$

The Specific Growth Rate (SGR) was also determined by applying the following formula [30]:

(3) $${SGR}_{j-i}\frac{\ln X_{j}-\ln X_{i}}{t_{j}-t_{i}}$$

where *X_i_* and *X_j_* represent the mean wet body weight for each diet at the beginning and end of the period, respectively, and *t_i_* and *t_j_* represent the time, in days, of the beginning and end of the period, respectively. Animals falling under the body condition score equal to two [31] were euthanized.

### 2.6. Morphological Analysis

At the end of the experimental period (when the animals were 44 weeks of age), the animals were euthanized with an overdose of MS-222 (0.3 mg/L). We collected gonads from *n* = 3 animals (female and male) per experimental group. By comparing the weight of the gonads with that of the whole animal, the Gonadosomatic Index (GSI) was calculated by using the following formula: (gonad weight/whole animal weight)∙100 [32]. Moreover, a head–trunk dissection was performed and fixed in Bouin’s solution for 24 h and then processed for paraffin embedding. Then, 7 μm thick sagittal sections of trunks were obtained using a Reichert–Jung 2050 microtome and then stained with hematoxylin-eosin (Bio-Optica Milano Spa, Milan, Italy. Code: 05-M06004), and observed and photographed under a Leica DM 1000 light microscope (Leica Microsystems, Wetzlar, Germany). Two different approaches for images’ collection were used for the males and females of each group, according to the different gonad sizes. For males, from each animal ten pics at 40× magnification of the gonad were captured. For females, three mosaics of the entire gonad, at a magnification of 10×, were generated per individual. To identify the gametes, we referred to the literature [33,34]. The counting was performed by using ImageJ software version 1.54j by three independent observers.

### 2.7. Statistical Analysis

The statistical analysis was performed using PRISM Version 10.2.3 (347). The Kaplan–Meier method was used to estimate animal survival.

A one-way ANOVA statistical analysis test, comparing the measured means of each group with the mean of every group, followed by multiple comparisons correction using the Tukey test, was conducted to assess the effects of the spirulina-supplemented diet. The difference between the groups was considered significant at a *p* value < 0.05.

As for BCI and SGR, the analysis was conducted by using a mixed-effects model followed by a Bonferroni correction, indicating BCI and SGR, respectively, as the dependent variables, and the fixed effects were chosen as “time” and “group” (representing different diets).

## 3. Results

### 3.1. Survival

**Figure 1:**
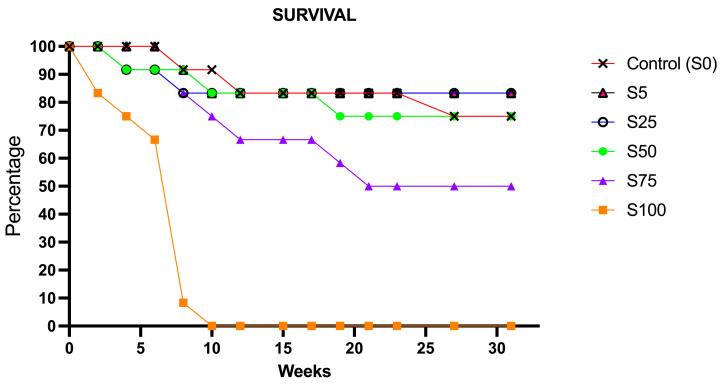
Survival rates of animals supplemented with spirulina at different percentages. The graph shows survival rates at the end of experimental period (32 weeks), equivalent to 75% for S0; 0% at week 10 for S100; 50% for S75; 70% for S50; 83.3% for S25; and 83.3% for S5.

The survival rate, recorded from week 0 to 32 of the experimental trial, is reported in Figure 1. The S0 group, which was fed only with standard food, showed a survival rate of 90% in week 10 and just over 80% in week 12. By the end of the experimental period, aint week 32, the survival rate for this group was around 75%. In the S100, receiving only spirulina as food source, individuals survived until week 10. At that time point all animals reached the humane endpoint [31] established in the project authorization and were euthanized. Specifically, the fish appeared markedly emaciated, showed a head wider than the trunk, and exhibited concave ventral and lateral abdominal margins. To avoid unnecessary suffering, all individuals were euthanized by anesthetic overdose (MS-222, 0.3 mg/L), according to the approved protocol. The S75, fed with 75% of spirulina, displayed a decrease over time, reaching a first peak at 12 weeks of the trial, when a survival of 67% was recorded, and a second peak at 22 weeks. Only 50% of the animals reached the end of the experimental period. In the S50, which received 50% of spirulina, three distinct peaks in mortality were observed: the first in week 4 with a 90% survival rate, the second in week 10 with approximately 80%, and the third in week 19, where survival approached 75%. The S25 and S5, which received 25% and 5% of spirulina, respectively, had a survival rate of 83.3% by week 10 and maintained constantly throughout the whole experimental period (Figure 1).

### 3.2. Body Measurements (Body Condition Index and Specific Growth Rate)

BCI is an index used to determine the health and nutritional status of an organism, indicating if it is well-conditioned and if it is receiving the appropriate amount of food to maintain a proper weight [29]. In zebrafish the ranges are as follows:

<0.8: Undernutrition;
Between 0.8 and 1.2: Good fitness.
>1.2: Overnutrition.

**Figure 2:**
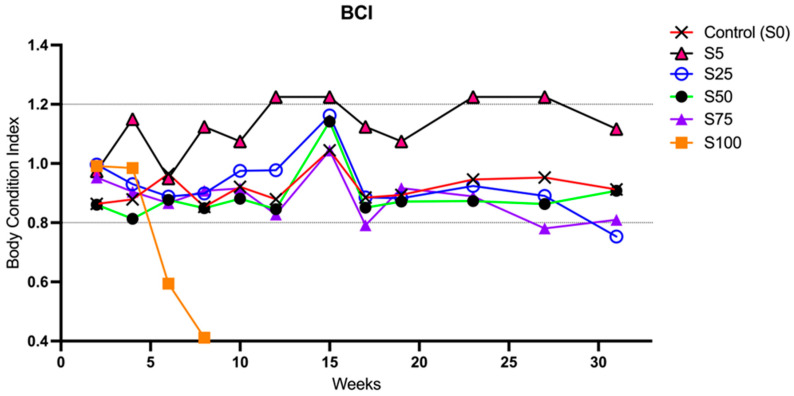
Longitudinal assessment of the Body Condition Index of animals supplemented with spirulina at different percentages. The graph shows data recorded from the first experimental week until week 32. The S0 group and groups S75, S50, and S25 fell into the recommended range (between 0.8 and 1.2) [29] suggestive of good fitness. The S100 group showed the drop from week 5 and S5 showed a slightly increased index at the week intervals 11–15 and 23–27.

At the end of the 32-week period, all fish demonstrated good fitness, remaining within the physiological range between 0.8 and 1.2 and showing a consistent trend throughout the trial (Figure 2). The BCI analysis also revealed that S5 exhibited a significantly higher BCI than other groups from the first week of the experimental period, although consistently within the optimal range. In contrast, S100 failed to reach the desired range and showed a rapid decline in BCI, falling into the suboptimal range. S0 and S25 displayed mean BCI values close to each other and generally higher than those displayed by the other groups (Figure 2).

**Figure 3:**
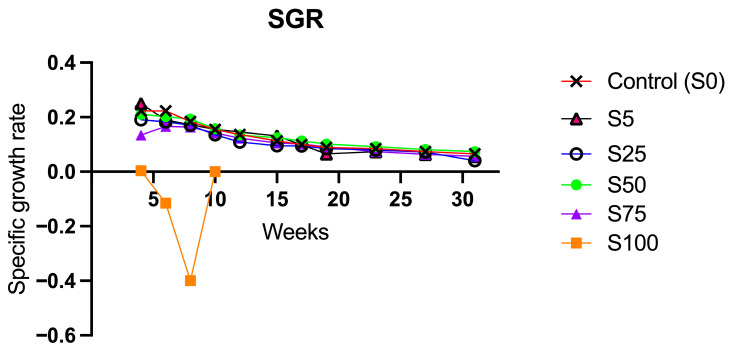
Longitudinal assessment of the Specific Growth Rates of animals supplemented with spirulina at different percentages. The graph shows data recorded from the first experimental week until week 32. All groups showed consistent physiological growth patterns which tend to decrease over time due to the adult-related slower growth. Only S100 displayed negative trend starting from week 4 of spirulina integration into the diet.

The SGR analysis did not show statistically significant differences among the experimental groups that completed the study compared to the S0 and was consistent with the literature [29] (Figure 3). The observed trend aligns with the expected physiological growth pattern of fish, where higher growth rates are noted at the beginning of the experimental period, followed by a gradual decrease towards the end of the period across all groups, except for S100. This trend likely reflects the natural reduction in growth rate as the animals age. Differing from the other groups, S100 exhibits a negative trend, appreciable from the second measurement, meaning that these fish started to lose weight instead of gaining it (Figure 3).

### 3.3. Reproductive and Spawning Performance

**Figure 4:**
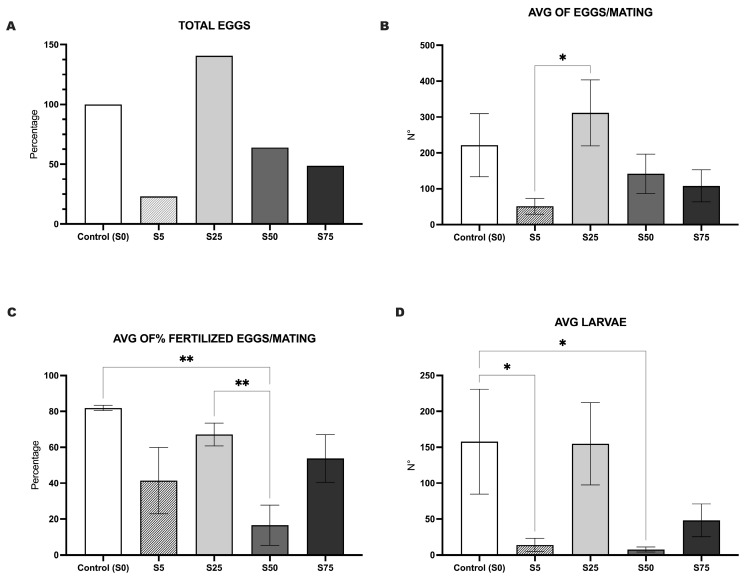
Reproductive fitness of animals supplemented with spirulina at different percentages. (**A**). The percentage of total eggs shows that at the end of experimental period, compared to the S0 group, the group S75 laid 48.6% of eggs, S50 63.9%, S25 140.5.%, and S5 23.1%. (**B**). The graph shows the average number of eggs laid per mating. S25 produced significantly more eggs than S5, and there were not any significant differences among the remaining Groups. (**C**). The graph indicates the percentage of fertilized eggs, showing the impact of diet on mother’s health. S0 and S25 had significantly higher percentages of fertilization rates than S50. There was no significant variation observed between the other groups. (**D**). The graph shows the average number of hatched larvae for each group. S0 and S25 produced significantly more larvae than S50 and S5, and did not have a significant difference with S75. Panels (**B**–**D**) represent the mean values obtained by averaging the results of all mating trials conducted for each experimental group The graph reports mean values ± standard deviation; * indicates a *p* value < 0.05 and ** indicates a *p* value < 0.01.

In terms of mating, compared to the S0 group, the group S25 laid a higher percentage of eggs, accounting for 140.5%. The other groups laid fewer eggs, equivalent to 63.9%, 48.6%, and 23.1% of eggs observed in S50, S75, and S5, respectively (Figure 4A). The average number of eggs laid per mating in each group, assessed by a comparison among experimental groups, revealed a significant difference between S25 and S5 (*p* value < 0.05) (Figure 4B). Any further significant differences were not observed among the other groups.

Our analysis of reproductive performance also examined the percentage of the fertilization rate of eggs, defined as the proportion of laid eggs that were successfully fertilized. This parameter is a key indicator of reproductive success, as it reflects both the quality of the diet and the overall health of the mothers. Eggs laid by nutritionally well-conditioned mothers are more likely to develop proper fertilization characteristics and successfully proceed through early development. Both S0 and S25 exhibited a significantly higher percentage of fertilized eggs compared to S50 (*p* value > 0.01). There were not any significant variations between or among the other groups (Figure 4C).

As the final indicator of reproductive performance, we assessed the average number of hatched larvae per group. The analysis revealed that, consistently with previous findings, S0 and S25 yielded the highest numbers of viable larvae, with an average of approximately 150 larvae. These groups showed significant differences compared to S50 and S5 (*p* value < 0.05), which exhibited notably reduced larval production. A significant difference was not observed when comparing S75 with either the S0 or S25 (Figure 4D). In Figures S1 and S2, we report the result evolution throughout the 32 weeks for eggs and larval production, respectively.

### 3.4. Gonadic Morphometric Analysis

**Figure 5:**
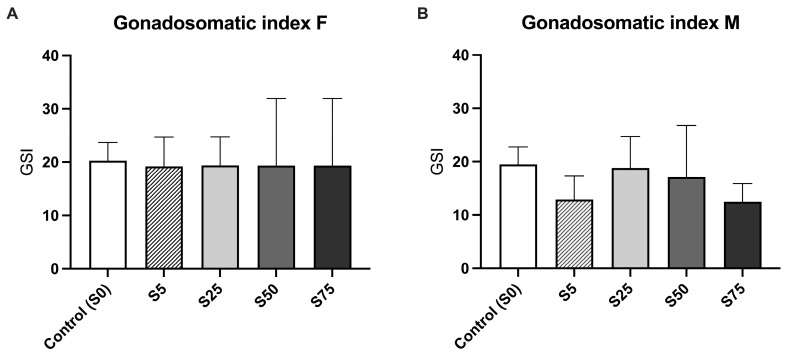
Analysis of the Gonadosomatic Index (GSI) (gonad weight/total body weight). (**A**). The Gonadosomatic Index in females did not show any significant differences among groups. (**B**). The Gonadosomatic Index in males did not show any significant differences among groups.

In addition to evaluating the effects of diets with different portions of spirulina on reproductive performance, a morphometric assessment of the gonads in both male and female animals was conducted in all experimental groups. The comparative analysis across the groups did not show any significant differences in GSI for either males or females (Figure 5A,B), indicating comparable reproductive stages among groups.

**Figure 6:**
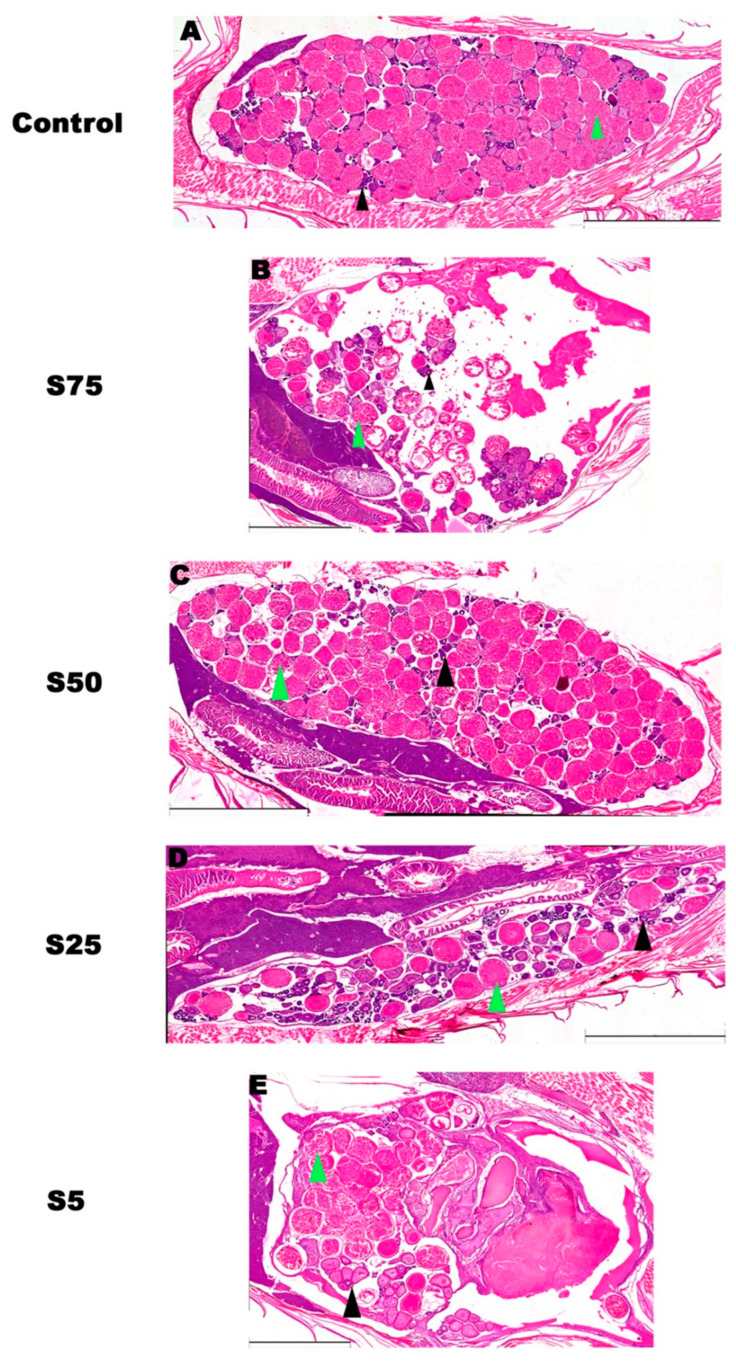
Mosaic of ovarian morphology of each experimental group. (**A**). S0 control group. (**B**). Group S75. (**C**). S50. (**D**). S25. (**E**). S5. Black arrowheads indicate pre-vitellogenic follicles, while green arrowheads indicate vitellogenic follicles. Scale bars: 2.8 mm in (**A**), 2.2 mm in (**B**), 3 mm in (**C**), 2.2 mm (**D**), and 2 mm in (**E**).

The morphometric parameters were based on the morphological identification of pre-vitellogenic and vitellogenic oocytes in females (Figure 6) and spermatogonia, spermatocytes, and spermatozoa in males (Figure 8).

**Figure 7:**
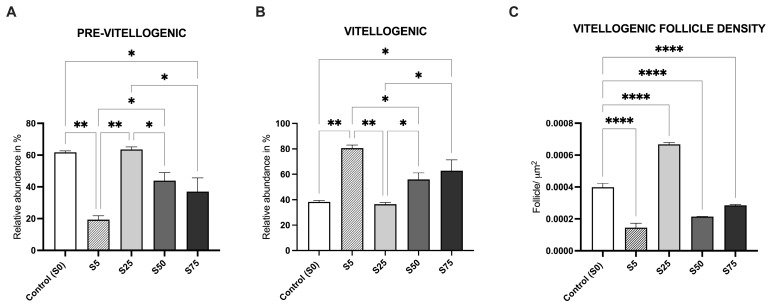
Morphological analysis of female gonads of animals supplemented with spirulina at different percentages. (**A**). Significantly higher pre-vitellogenic oocytes abundance in S0 control and in the S25 group compared to groups S75, S50, and S5. (**B**). Significantly higher vitellogenic oocytes abundance in S75, S50, and S5 compared to S0 control and the S25 group. (**C**). Vitellogenic follicle density (calculated number of vitellogenic oocytes/the ovarian area) was higher in S0 control and the S25 group compared to the others. The graph reports mean values ± standard deviation; * indicates a *p* value < 0.05, ** indicates a *p* value < 0.01, and **** indicates a *p* value < 0.001.

For females, data indicated that the relative abundance (%) of pre-vitellogenic oocytes was the highest in the S0 and S25 groups, and this was significantly higher than in groups S75 (*p* value < 0.05), S50 (*p* value < 0.01), and S5 (*p* value < 0.01) (Figure 7A). The relative abundance of vitellogenic oocytes was higher in S75, S50, and S5 compared to the S0 and S25 groups, with the highest values in groups S75 and S5 (Figure 7B). Finally, the vitellogenic follicle density, evaluated by comparing the ratio between the number of vitellogenic oocytes and the ovarian area, revealed the highest significant density in the S0 and S25 groups compared to S75, S50, and S5 (*p* value < 0.001) (Figure 7C).

**Figure 8:**
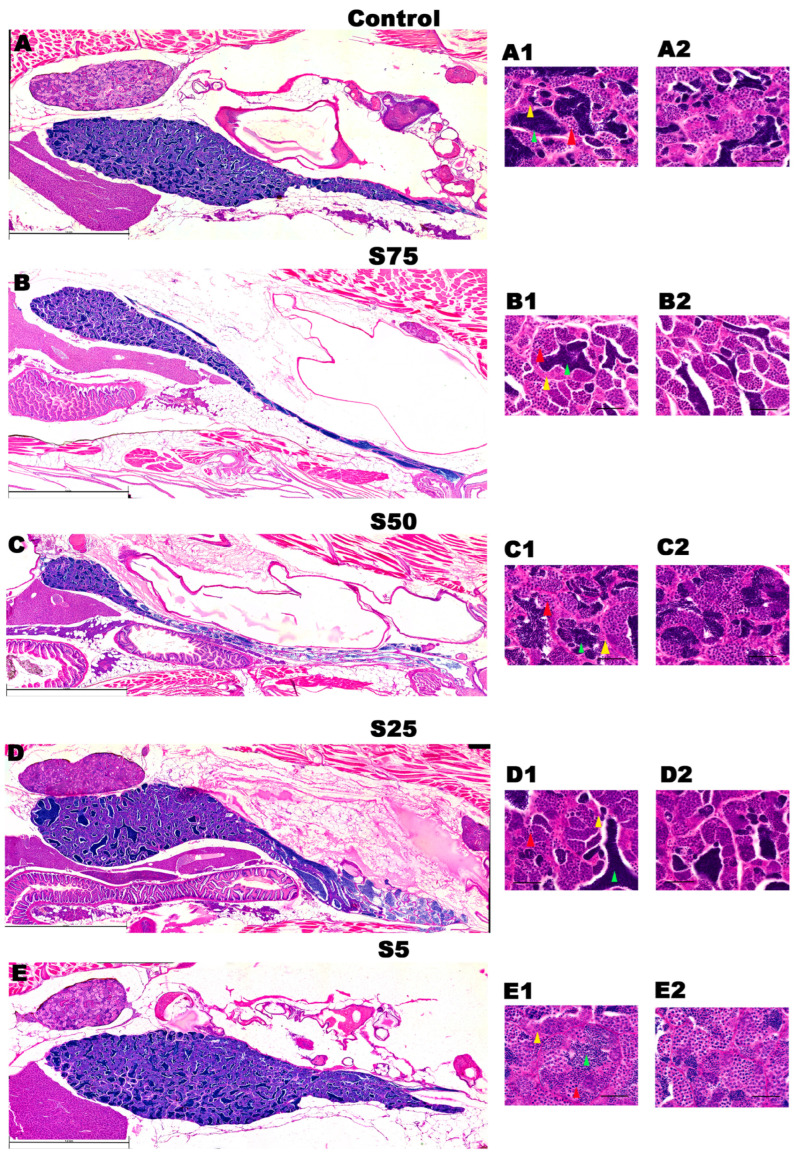
Mosaic of testis morphology of each experimental group. (**A**). Overview of S0 control male testis and higher magnification in (**A1**,**A2**). (**B**). Overview of S75 male testis and higher magnification in (**B1**,**B2**). (**C**). Overview of S50 male testis and higher magnification in (**C1**,**C2**). (**D**). Overview of S25 male testis and higher magnification in (**D1**,**D2**). (**E**). Overview of S5 male testis and higher magnification in (**E1**,**E2**). Yellow arrowheads indicate spermatogonia, red arrowheads indicate spermatocytes, and green arrowhead spermatozoa. Scale bars: 1.4 mm in (**A**), 1.6 mm in (**B**), 1.4 mm in (**C**), 1.6 mm in (**D**), 1.2 mm in (**E**), and 50 μm in all the inlets.

**Figure 9:**
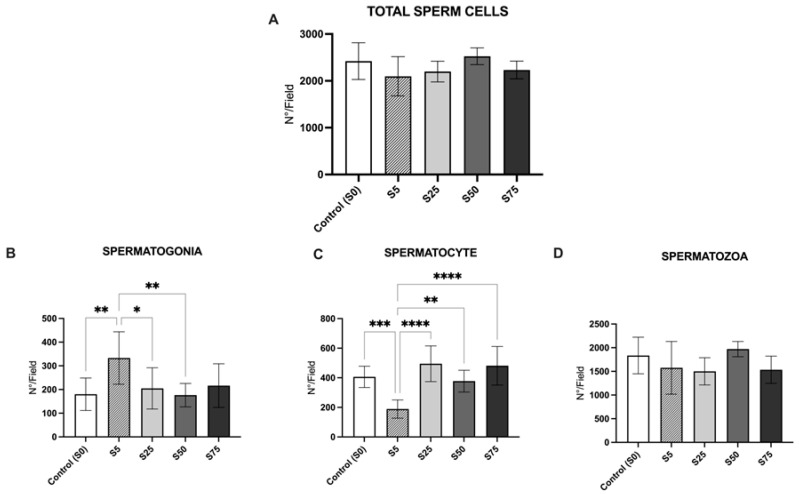
Morphological analysis of male gonads of animals supplemented with spirulina at different percentages. (**A**). No significant difference was observed among all groups. (**B**). Significantly higher abundance of spermatogonia in S5 compared to S0, S50, and S25 groups. (**C**). Significantly lower abundance of spermatocytes in S5 compared to all other groups. (**D**). Any significant difference was not observed in the spermatozoa counting among all groups. The graph reports mean values ± standard deviation; * indicates a *p* value < 0.05, ** indicates a *p* value < 0.01, *** indicates a *p* value < 0.005, and **** indicates a *p* value < 0.001.

For males, in Figure 8 are presented mosaic of testis morphology representative of each experimental group. According to our analysis all groups displayed a comparable number of total sperm cells per field, and there were with not any significant differences among them (Figure 9A). Significant differences were observed in the spermatogonia counting, with S5 showing a significantly higher number of spermatogonia compared to all other groups (*p* value < 0.01) (Figure 9B). S5 displayed the lowest number of spermatocytes, with a significant reduction observed compared to all other groups (*p* value < 0.001) (Figure 9C). Finally, all groups displayed a comparable number of spermatozoa (Figure 9D) indicating that, despite variations in earlier germ cell stages, the final number of mature sperm cells remained consistent.

## 4. Discussion

The combination of high-quality proteins, essential fatty acids, a diverse range of vitamins, and bioactive pigments, make spirulina a remarkable functional ingredient with potential applications in nutrition, medicine, and beyond. Its nutrient density and health-promoting properties continue to position it as a subject of significant interest in the scientific community; as proof, some authors have proposed spirulina to partially or completely replace protein sources in the diets of many farm animals [35,36,37]. However, in aquatic animals, including both farmed and ornamental fish species as well as shrimps, it has been used as a dietary supplement to aquafeeds with varying supplementation/substitution percentages [38].

### 4.1. Effects of Spirulina on Survival and Growth Performance

Previous studies have shown that the effects of spirulina supplementation vary widely among fish species. In Nile tilapia (*Oreochromis niloticus*), Belal et al. (2012) reported improved Specific Growth Rates with 1% spirulina inclusion [39]. Other studies found enhanced growth at 30% *A. platensis*, no effect at 60%, and reduced growth at 75% supplementation [40]. Feeding tilapia with 100% raw *A. platensis* in a closed recirculating system caused no visible larval abnormalities [41]. Trials on tilapia hybrids yielded inconsistent results: *A. maxima* (20–100%) had no effect, while 75% *A. platensis* improved fitness [42,43]. In carp, spirulina (25–100%) did not enhance performance [44].

Similarly variable responses were observed in other species, including guppies, *Xiphophorus helleri*, sturgeon (*Acipenser oxyrinchus*), seabream (*Rhabdosargus sarba*), and catfish (*Clarias gariepinus*). The benefits were often dose and species dependent, such as improved growth at 10% in sturgeon [45] and 8% in *Xiphophorus* [46], whereas no positive effects and even growth inhibition at 75% were noted in silver seabream [47]. Additionally, *A. platensis* supplementation has been shown to mitigate the adverse effects of hydroxychloroquine [48] and gibberellic acid [49] in fish.

Thanks to its rich nutrient content, dietary spirulina has been shown to support fish homeostasis through multiple mechanisms. Studies in zebrafish have demonstrated its antioxidant [50], microbiota-sustaining [51], neuroprotective and anxiolytic, immune-modulatory [52], and wound-healing [53] effects. Consistent with these findings, our data revealed higher survival in fish fed spirulina-supplemented diets compared to controls. Over 32 weeks, the control and S50 groups showed 75% survival, while S100 reached humane endpoints by week 10, indicating detrimental effects of an exclusively spirulina diet. Conversely, S25 and S5 achieved the best outcomes, maintaining ~83% survival throughout. These results suggest that moderate integration (5–25%) within a balanced diet optimally supports long-term health, whereas excessive inclusion leads to nutritional imbalance and reduced viability.

To assess the effects of spirulina integration, we measured both the Specific Growth Rate (SGR) and the Body Condition Index (BCI), the latter being a practical, non-invasive indicator of fish nutritional and physiological status [54]. While SGR did not differ significantly among diets, BCI showed clear variation. Fish fed the standard diet supplemented with 5% *A. platensis* displayed higher BCI values from week 7 onward, indicating improved overall health. No notable differences were observed in other groups except S100, in which complete diet replacement with *A. platensis* impaired growth and led to early study termination due to humane endpoints [31]. The early decline of the S100 group and the necessity to euthanize all animals upon reaching predefined humane endpoints indicate that a diet consisting of 100% spirulina is incompatible with long-term health in zebrafish and ethically unacceptable. Furthermore, such an unbalanced dietary regimen has no realistic applicability in aquaculture practice, where spirulina is employed exclusively as a dietary supplement rather than as a sole feed source. A critical observation raised regarding the intervention point for euthanasia involves the severity of clinical signs, such as marked emaciation, observed in the S100 group. While we acknowledge that these signs reflected a state of physiological stress, it is crucial to emphasize that each animal within all the experimental groups were daily monitored and the humane endpoints—including specific body condition scores—were rigorously defined a priori and were integral to the experimental protocol approved by the ethical committee. Adherence to these pre-established criteria was essential for the scientific validity of the study, as the primary objective was to accurately evaluate the full spectrum of effects caused by progressive spirulina substitution and to determine the maximum inclusion level impacting survival. Therefore, reaching the planned endpoint was necessary to provide a comprehensive and correct nutritional assessment.

The outcomes are scientifically valuable because they delineate the upper biological limits of spirulina inclusion and provide critical information to avoid inappropriate feeding strategies that could compromise fish welfare. Many studies have reported the positive effects of spirulina on fish growth. In our study, growth performance remained stable despite increasing spirulina levels, likely reflecting species-specific digestive morphology and function [55,56]. These findings underscore the need to optimize integration rates to achieve benefits without compromising health or productivity.

### 4.2. Modulation of Reproduction by Dietary Spirulina

Beyond its effects on growth and survival, our study demonstrates that diet composition, specifically spirulina integration, significantly influences reproductive physiology in zebrafish.

#### 4.2.1. Spawning Performance

The superior reproductive performance of the S25 group likely reflects enhanced reproductive behavior supported by optimal nutritional balance. Adequate and well-balanced fatty acid intake is known to modulate neuroendocrine pathways governing reproduction in fish [10,11]. The reduced spawning activity at higher spirulina levels (50–75%) suggests that excessive integration may disrupt metabolic or hormonal homeostasis. Conversely, the limited output in the S5 group, despite their good survival rates and condition, indicates that minimal integration may not fully sustain reproductive performance.

#### 4.2.2. Quantitative Reproductive Outcomes

The S25 group showed a 140% increase in egg production, higher fertilization rates, and greater larval viability compared to S0. These outcomes align with previous studies linking spirulina’s bioactive components, such as essential fatty acids and carotenoids, to enhanced reproductive performance in fish [54]. The similar fertilization rates in S25 and S0 indicate that moderate integration sustains reproductive efficiency while improving overall output. Overall, a 25% spirulina inclusion appears optimal for supporting gamete quality, spawning activity, and early larval development.

### 4.3. Gonadal Development and Gametogenesis

The morphometric analysis of the gonads complemented reproductive data, linking performance to tissue condition. The Gonadosomatic Index (GSI), an indicator of sexual maturity [57], showed no significant differences between groups in either sex, suggesting comparable gonadal development across diets.

#### 4.3.1. Female Gonadal Development

A detailed morphometric analysis of female gonads focused on the relative abundance of pre-vitellogenic and vitellogenic oocytes and on follicle density, key indicators of oocyte maturation in fish [58]. Pre-vitellogenic oocytes were most abundant in S0 and S25, with significantly higher values than in S75, S50, and S5, indicating that 25% spirulina supports early oocyte development similarly to the control diet. Conversely, vitellogenic oocytes were significantly more abundant in S75, S50, and S5, suggesting that higher or lower spirulina levels may accelerate oocyte maturation compared to the standard diet. The analysis of vitellogenic follicle density, expressed as the ratio of vitellogenic oocytes to the ovarian area, further clarified the effects of spirulina integration. The S25 group showed the highest density, while S75, S50, and S5 exhibited significantly lower values. The superior performance of the 25% spirulina diet may be partly explained by its balanced supply of fatty acids, particularly omega-3 and omega-6 polyunsaturated fatty acids (PUFAs). These nutrients are known to modulate membrane fluidity, hormonal synthesis, and signaling pathways involved in oocyte maturation and reproductive behavior. An optimal omega-3/omega-6 ratio likely supports proper neuroendocrine and metabolic function, contributing to the enhanced gonadal development and reproductive output observed in the S25 group.

#### 4.3.2. Male Gonadal Development

The differences in germ cell composition likely reflect the nutritional and bioactive profile of spirulina, which supports spermatogenesis through antioxidant, metabolic, and endocrine pathways. Moderate integration (25%) may provide an optimal balance of essential amino acids, vitamins, carotenoids, and polyunsaturated fatty acids—particularly omega-3 and omega-6—enhancing hormonal regulation and promoting the progression of spermatogonia to spermatocytes. In contrast, low integration (5%) may stimulate early proliferation but fail to sustain maturation due to limited nutrient availability. Similar effects of spirulina on testicular function and sperm quality have been reported in zebrafish, tilapia, and catfish, emphasizing its role in redox balance and steroidogenesis [59,60].

### 4.4. Nutritional Mechanisms Underlying Reproductive Effects

The differential outcomes observed among dietary groups further emphasize the dose-dependent effects of spirulina integration. *A. platensis* provides a highly nutritious profile, rich in proteins (60–70%), essential amino acids, polyunsaturated fatty acids (notably omega-3 and omega-6), vitamins (B-complex and E), minerals (iron, zinc, calcium, and magnesium), and bioactive compounds such as carotenoids and phycocyanin. These nutrients are fundamental to sustaining the elevated energetic demands of gametogenesis [61]. In particular, fatty acids play a critical role in reproductive physiology. Jaya-Ram et al. [62] demonstrated that an optimal n-3/n-6 HUFA ratio supports zebrafish reproduction by providing adequate precursors (18:2n-6, 18:3n-3), whereas excessive levels impair performance. Maintaining this balance ensures sufficient DHA, EPA, and ARA availability for gamete maturation and embryonic development. Accordingly, the enhanced reproductive performance observed at 25% spirulina integration likely reflects an optimal equilibrium between nutrient availability, antioxidant capacity, and energy metabolism, supporting both oogenesis and spermatogenesis. In contrast, the 5% integration rate appears inadequate to meet these metabolic demands, resulting in limited germ cell maturation and reduced reproductive output.

## 5. Conclusions

This longitudinal study demonstrates that spirulina integration exerts clear dose-dependent effects on zebrafish survival, growth, health, and reproduction. Integration at 5% and 25%, combined with a balanced diet, produced distinct yet beneficial outcomes. A 5% inclusion supports optimal body condition, longevity, and general welfare, while 25% markedly enhances reproductive behavior, gonadal development, and offspring production, identifying it as the optimal level for breeding programs. These findings support a precision nutrition strategy in aquaculture, tailoring spirulina levels to specific life stages. Moreover, the improved reproductive metrics at 25% likely reflect synergistic effects on both the physiological and behavioral components of reproduction, underscoring spirulina’s potential to enhance reproductive efficiency, welfare, and sustainability in fish culture. Given that the SDS 400 standard diet is specifically recommended for boosting growth and conditioning adults for spawning, the additional improvement observed with spirulina integration highlights its strong functional potential. Such an effect of a high-performance diet suggests that even greater benefits could be achieved with other commercial feeds.

## Figures and Tables

**Table 1:** SDS nutritional profile and comparison with spirulina.

	SDS	Spirulina
Proteins	60 g/100 g	62 g/100 g
Lipid	14.5 g/100 g	6.3 g/100 g
Carbohydrate	11 g/100 g	10 g/100 g
Fiber	3 g/100 g	1.6 g/100 g
Ashes	11.5 g/100 g	6.8 g/100 g

**Table 2:** Spirulina’s nutritional profile as provided by M2M Engineering (Naples, Italy).

NUTRITIONALS VALUES			
Calories	353 kcal/100 g	Amino acid profile	
Moisture	4.14 g/100 g	Alanine	4.45 g/100 g
Ashes	6.8 g/100 g	Arginine	3.56 g/100 g
Macronutrients		Aspartic acid	5.18 g/100 g
Proteins	62 g/100 g	Glutamic acid	7.36 g/100 g
Carbohydrate	10 g/100 g	Glycine	2.66 g/100 g
Sugar	1.3 g/100 g	Histidine	0.807 g/100 g
Fatty acids	6.3 g/100 g	Hydroxyproline	0.2 g/100 g
Saturated	2.4 g/100 g	Isoleucine	3.01 g/100 g
Monounsaturated	0.7 g/100 g	Leucine	4.73 g/100 g
Polynsaturated	3.2 g/100 g	Lysine	2.33 g/100 g
Omega-6	1.6 g/100 g	Ornithine	0.05 g/100 g
Omega-3	1.6 g/100 g	Phenylalanine	2.46 g/100
Fiber	1.6 g/100 g	Proline	1.88 g/100
Micronutrients		Serine	2.76 g/100
Calcium	124 mg/100 g	Threonine	2.75 g/100
Phosphorus	647 mg/100 g	Tyrosine	2.53 g/100 g
Magnesium	168 mg/100 g	Valine	3.23 g/100 g
Iron	51 mg/100 g	Cystein + cystine	0.493 g/100 g
Zinc	17.6 mg/100 g	Methionine	1.36 g/100 g
Manganese	2.99 mg/100 g	Tryptophan	0.869 g/100 g
Copper	6.8 mg/100 g		
Iodine	74 mg/100 g	Phytopigments	
Sodium	705 mg/100 g	Phycocyanin	12.80%
Potassium	1780 mg/100 g	C-phycocyanin	13.00%
Beta carotenoid	87.9 mg/100 g	Chlorophyll a	2.1 mg/100 g
Vitamin b1	0.3 mg/100 g	Carotenoids	87.4 mg/100 g
Vitamin b2	0.37 mg/100 g	N-p-k-c	
Vitamin b3	2.11 mg/100 g	N-nitrogen	8.8 g/100 g
Vitamin b6	0.16 mg/100 g	P-phosphorous	0.64 g/100 g
Vitamin b12	0.025 mg/100 g	K-potassium	1.78 g/100 g
Vitamin a	0.033 mg/100 g	C-carbon	46.4 g/100 g
Vitamin e	12.3 mg/100 g	Nucleic acids	
Vitamin h	0.025 mg/100 g	Nucleic acids DNA	120 mg/100 g

## Data Availability

Dataset available on request from the authors.

## Supplementary Materials

The following supporting information can be downloaded at: https://www.mdpi.com/article/10.3390/ani16010098/s1. Figure S1: Deposed eggs over time; Figure S2: Hatched larvae over time; Table S1:Different diet composition.

## References

[1] Batista A.P., Nunes M.C., Fradinho P., Gouveia L., Sousa I., Raymundo A., Franco J.M. (2012). Novel Foods with Microalgal Ingredients—Effect of Gel Setting Conditions on the Linear Viscoelasticity of Spirulina and Haematococcus Gels. J. Food Eng..

[2] Becker E.W. (2007). Micro-Algae as a Source of Protein. Biotechnol. Adv..

[3] Vonshak A. (2014). Spirulina Platensis Arthrospira: Physiology, Cell-Biology and Biotechnology.

[4] El-Moataaz S., Ismael H., Aborhyem S. (2019). Assessment of Chemical Composition of Spirulina Platensis and Its Effect on Fasting Blood Glucose and Lipid Profile in Diabetic Rats. J. High Inst. Public Health.

[5] Parrish C.C., Kainz M., Brett M.T., Arts M.T. (2009). Essential Fatty Acids in Aquatic Food Webs. Lipids in Aquatic Ecosystems.

[6] Masuda K., Chitundu M. (2019). Multiple Micronutrient Supplementation Using Spirulina Platensis during the First 1000 Days Is Positively Associated with Development in Children under Five Years: A Follow up of a Randomized Trial in Zambia. Nutrients.

[7] Kumar A., Ramamoorthy D., Verma D.K., Kumar A., Kumar N., Kanak K.R., Marwein B.M., Mohan K. (2022). Antioxidant and Phytonutrient Activities of Spirulina Platensis. Energy Nexus.

[8] Athiyappan K.D., Routray W., Paramasivan B. (2024). Phycocyanin from Spirulina: A Comprehensive Review on Cultivation, Extraction, Purification, and Its Application in Food and Allied Industries. Food Humanit..

[9] Ilieva Y., Zaharieva M.M., Najdenski H., Kroumov A.D. (2024). Antimicrobial Activity of Arthrospira (Former Spirulina) and Dunaliella Related to Recognized Antimicrobial Bioactive Compounds. Int. J. Mol. Sci..

[10] Volkoff H., London S. (2018). Nutrition and Reproduction in Fish. Encyclopedia of Reproduction.

[11] Assan D., Huang Y., Mustapha U.F., Addah M.N., Li G., Chen H. (2021). Fish Feed Intake, Feeding Behavior, and the Physiological Response of Apelin to Fasting and Refeeding. Front. Endocrinol..

[12] Li W., Du R., Xia C., Zhang H., Xie Y., Gao X., Ouyang Y., Yin Z., Hu G. (2022). Novel Pituitary Actions of GnRH in Teleost: The Link between Reproduction and Feeding Regulation. Front. Endocrinol..

[13] Carneiro W.F., Navarrete-Ramírez P., Castro T.F.D., Leão E.R., Martínez-Chávez C.C., Martínez-Palacios C.A., Murgas L.D.S. (2025). Replacing Fish Meal with Spirulina (*Arthrospira platensis*): Nutrigenomic Modulation of Growth, Reproductive Performance, and Metabolism in Zebrafish. Animals.

[14] Jardine D., Litvak M.K. (2003). Direct Yolk Sac Volume Manipulation of Zebrafish Embryos and the Relationship between Offspring Size and Yolk Sac Volume. J. Fish Biol..

[15] Yilmaz O., Patinote A., Com E., Pineau C., Bobe J. (2021). Knock out of Specific Maternal Vitellogenins in Zebrafish (*Danio rerio*) Evokes Vital Changes in Egg Proteomic Profiles That Resemble the Phenotype of Poor Quality Eggs. BMC Genom..

[16] Cacialli P., Gueguen M.-M., Coumailleau P., D’Angelo L., Kah O., Lucini C., Pellegrini E. (2016). BDNF Expression in Larval and Adult Zebrafish Brain: Distribution and Cell Identification. PLoS ONE.

[17] Ulloa P.E. (2014). Zebrafish as Animal Model for Aquaculture Nutrition Research. Front. Genet..

[18] Aleström P., D’Angelo L., Midtlyng P.J., Schorderet D.F., Schulte-Merker S., Sohm F., Warner S. (2020). Zebrafish: Housing and Husbandry Recommendations. Lab. Anim..

[19] Westerfield M., Book T. (2000). A Guide for the Laboratory Use of Zebrafish Danio (Brachydanio) Rerio.

[20] Reading B.J., Andersen L.K., Ryu Y.-W., Mushirobira Y., Todo T., Hiramatsu N. (2018). Oogenesis and Egg Quality in Finfish: Yolk Formation and Other Factors Influencing Female Fertility. Fishes.

[21] Benini E., Politis S.N., Nielsen A., Sørensen S.R., Tomkiewicz J., Engrola S. (2022). Type of Hormonal Treatment Administered to Induce Vitellogenesis in European Eel Influences Biochemical Composition of Eggs and Yolk-Sac Larvae. Fish Physiol. Biochem..

[22] Howe D.G., Bradford Y.M., Eagle A., Fashena D., Frazer K., Kalita P., Mani P., Martin R., Moxon S.T., Paddock H. (2017). The Zebrafish Model Organism Database: New Support for Human Disease Models, Mutation Details, Gene Expression Phenotypes and Searching. Nucleic Acids Res..

[23] Lawrence C. (2007). The Husbandry of Zebrafish (*Danio rerio*): A Review. Aquaculture.

[24] Abd El-Rahman G.I. (2018). Evaluation the Efficacy of Combined Mixture of Spirulina Platensisand Cinnamon Extracts in Overweight Rats Fed on a Fatty Diet. Life Sci. J..

[25] Remirez D., González R., Merino N., Rodriguez S., Ancheta O. (2002). Inhibitory Effects of Spirulina in Zymosan-induced Arthritis in Mice. Mediat. Inflamm..

[26] Torres-Durán P.V., Ferreira-Hermosillo A., Ramos-Jiménez A., Hernández-Torres R.P., Juárez-Oropeza M.A. (2012). Effect of Spirulina Maxima on Postprandial Lipemia in Young Runners: A Preliminary Report. J. Med. Food.

[27] Hillman C., Cooper A.H., Ram P., Parker M.O. (2024). The Effect of Laboratory Diet and Feeding on Growth Parameters in Juvenile Zebrafish. Lab Anim..

[28] Avdesh A., Chen M., Martin-Iverson M.T., Mondal A., Ong D., Rainey-Smith S., Taddei K., Lardelli M., Groth D.M., Verdile G. (2012). Regular Care and Maintenance of a Zebrafish (*Danio rerio*) Laboratory: An Introduction. J. Vis. Exp. JoVE.

[29] Fowler L.A., Williams M.B., Dennis-Cornelius L.N., Farmer S., Barry R.J., Powell M.L., Watts S.A. (2019). Influence of Commercial and Laboratory Diets on Growth, Body Composition, and Reproduction in the Zebrafish *Danio rerio*. Zebrafish.

[30] Brinker A., Reiter R. (2011). Fish Meal Replacement by Plant Protein Substitution and Guar Gum Addition in Trout Feed, Part I: Effects on Feed Utilization and Fish Quality. Aquaculture.

[31] Clark T.S., Pandolfo L.M., Marshall C.M., Mitra A.K., Schech J.M. (2018). Body Condition Scoring for Adult Zebrafish (*Danio rerio*). J. Am. Assoc. Lab. Anim. Sci..

[32] Schiano V., Cutignano A., Maiello D., Carbone M., Ciavatta M., Polese G., Fioretto F., Attanasio C., Palladino A., Felline S. (2022). An Alkaloid from a Highly Invasive Seaweed Increases the Voracity and Reproductive Output of a Model Fish Species. Mar. Drugs.

[33] Menke A.L., Spitsbergen J.M., Wolterbeek A.P.M., Woutersen R.A. (2011). Normal Anatomy and Histology of the Adult Zebrafish. Toxicol. Pathol..

[34] Petrovici A., Popovici I., Solcan C. (2017). Histological Structure of the Testis in Adult Zebrafish (*Danio rerio*). https://repository.iuls.ro/xmlui/handle/20.500.12811/1243.

[35] Altmann B.A., Rosenau S. (2022). Spirulina as Animal Feed: Opportunities and Challenges. Foods.

[36] Lambiase C., Braghieri A., Barone C.M.A., Di Francia A., Pacelli C., Serrapica F., Lorenzo J.M., De Rosa G. (2023). Use of Cyanobacterium Spirulina (*Arthrospira platensis*) in Buffalo Feeding: Effect on Mozzarella Cheese Quality. Foods.

[37] Waheed D.M., El-Diasty M., Gabr E.M. (2024). Spirulina as an Animal Feed and Its Effect on Animal Health and Productivity. J. Adv. Vet. Res..

[38] Rosas V.T., Poersch L.H., Romano L.A., Tesser M.B. (2019). Feasibility of the Use of *Spirulina* in Aquaculture Diets. Rev. Aquac..

[39] Belal E.B., Khalafalla M., El-Hais A. (2012). Use of Spirulina (*Arthrospira fusiformis*) for Promoting Growth of Nile Tilapia Fingerlings. Afr. J. Microbiol. Res..

[40] Velasquez S.F., Chan M.A., Abisado R.G., Traifalgar R.F.M., Tayamen M.M., Maliwat G.C.F., Ragaza J.A. (2016). Dietary Spirulina (*Arthrospira platensis*) Replacement Enhances Performance of Juvenile Nile Tilapia (*Oreochromis niloticus*). J. Appl. Phycol..

[41] Takeuchi T., Lu J., Yoshizaki G., Satoh S. (2002). Effect on the Growth and Body Composition of Juvenile Tilapia Oreochromis Niloticus Fed Raw Spirulina. Fish. Sci..

[42] El-Sheekh M., El-Shourbagy I., Shalaby S., Hosny S. (2014). Effect of Feeding *Arthrospira platensis* (Spirulina) on Growth and Carcass Composition of Hybrid Red Tilapia (*Oreochromis niloticus* x *Oreochromis mossambicus*). Turk. J. Fish. Aquat. Sci..

[43] Kamal A.H.M.M., Mair G.C. (2005). Salinity Tolerance in Superior Genotypes of Tilapia, *Oreochromis niloticus*, *Oreochromis mossambicus* and Their Hybrids. Aquaculture.

[44] Nandeesha M., Gangadhara B., Manissery J., Venkataraman L. (2001). Growth Performance of Two Indian Major Carps, Catla (Catlacatla) and Rohu (Labeorohita) Fed Diets Containing Different Levels of Spirulina Platensis. Bioresour. Technol..

[45] Adel M., Yeganeh S., Dadar M., Sakai M., Dawood M.A.O. (2016). Effects of Dietary Spirulina Platensis on Growth Performance, Humoral and Mucosal Immune Responses and Disease Resistance in Juvenile Great Sturgeon (*Huso huso* Linnaeus, 1754). Fish Shellfish Immunol..

[46] James R., Sampath K., Thangarathinam R., Vasudevan I. (2006). Effect of Dietary Spirulina Level on Growth, Fertility, Coloration and Leucocyte Count in Red Swordtail, Xiphophorus Helleri. Isr. J. Aquac.-Bamidgeh.

[47] El-Sayed A.-F.M. (1994). Evaluation of Soybean Meal, Spirulina Meal and Chicken Offal Meal as Protein Sources for Silver Seabream (*Rhabdosargus sarba*) Fingerlings. Aquaculture.

[48] Sayed A.E.-D.H., Hamed M., Soliman H.A. (2021). Spirulina Platensis Alleviated the Hemotoxicity, Oxidative Damage and Histopathological Alterations of Hydroxychloroquine in Catfish (*Clarias gariepinus*). Front. Physiol..

[49] Sayed A.E.-D.H., Hamed M., El-Sayed A.A., Nunes B., Soliman H.A. (2023). The Mitigating Effect of Spirulina (*Arthrospira platensis*) on the Hemotoxicity of Gibberellic Acid on Juvenile Tilapia (*Oreochromis niloticus*). Environ. Sci. Pollut. Res..

[50] Coli A.P., Carneiro W.F., Da Silva K.C.D., Castro T.F.D., De Oliveira J.P.L., De Martins M.S.A., Murgas L.D.S. (2024). Spirulina (*Arthrospira platensis*) Supplementation: Impact on Growth, Metabolism, and Antioxidant Status in Zebrafish. Anim. Physiol. Nutr..

[51] Ma K., Chen S., Wu Y., Ma Y., Qiao H., Fan J., Wu H. (2022). Dietary Supplementation with Microalgae Enhances the Zebrafish Growth Performance by Modulating Immune Status and Gut Microbiota. Appl. Microbiol. Biotechnol..

[52] Edirisinghe S.L., Dananjaya S.H.S., Nikapitiya C., Liyanage T.D., Lee K.-A., Oh C., Kang D.-H., De Zoysa M. (2019). Novel Pectin Isolated from Spirulina Maxima Enhances the Disease Resistance and Immune Responses in Zebrafish against Edwardsiella Piscicida and Aeromonas Hydrophila. Fish Shellfish Immunol..

[53] Noh H.-J., Son H.-Y. (2020). Effect of Spirulina on Corneal Epithelial Wound Healing in Zebrafish. J. Prev. Veter-Med..

[54] Brosset P., Averty A., Mathieu-Resuge M., Schull Q., Soudant P., Lebigre C. (2023). Fish Morphometric Body Condition Indices Reflect Energy Reserves but Other Physiological Processes Matter. Ecol. Indic..

[55] De Felice E., Palladino A., Tardella F.M., Giaquinto D., Barone C.M.A., Crasto A., Scocco P. (2021). A Morphological, Glycohistochemical and Ultrastructural Study on the Stomach of Adult Rainbow Trout *Oncorhynchus mykiss*. Eur. Zool. J..

[56] Palladino A., De Felice E., Attanasio C., Barone C.M.A., Crasto A., D’Angelo L., Giaquinto D., Lambiase C., Scocco P., Serrapica F. (2023). A Morphological and Ultrastructural Study of the Anterior Digestive Tract of Adult Nile Tilapia Oreochromis Niloticus. Animals.

[57] Flores A., Wiff R., Ganias K., Marshall C.T. (2019). Accuracy of Gonadosomatic Index in Maturity Classification and Estimation of Maturity Ogive. Fish. Res..

[58] Mahalingam A., Santhanam P. (2023). Vitellogenesis and Reproductive Strategies in Fishes. Vitellogenin in Fishes-Diversification, Biological Properties, and Future Perspectives.

[59] Mabrouk M.M., Ashour M., Younis E.M., Abdel-Warith A.-W.A., Bauomi M.A., Toutou M.M., Mansour A.I., Abdelaty B.S., Elokaby M.A., Davies S.J. (2024). Arthrospira Platensis Nanoparticles Dietary Supplementation Improves Growth Performance, Steroid Hormone Balance, and Reproductive Productivity of Nile Tilapia (*Oreochromis niloticus*) Broodstock. PLoS ONE.

[60] Zhang Y., Zhou Y., Tang Q., Hu F., Feng L., Shen J., Huang B. (2018). The Protective Effects of Selenium-Enriched Spirulina on the Reproductive System of Male Zebrafish (*Danio rerio*) Exposed to Beta-Cypermethrin. Food Funct..

[61] Marvel M., Levavi-Sivan B., Wong T.-T., Zmora N., Zohar Y. (2021). Gnrh2 Maintains Reproduction in Fasting Zebrafish through Dynamic Neuronal Projection Changes and Regulation of Gonadotropin Synthesis, Oogenesis, and Reproductive Behaviors. Sci. Rep..

[62] Jaya-Ram A., Kuah M.-K., Lim P.-S., Kolkovski S., Shu-Chien A.C. (2008). Influence of Dietary HUFA Levels on Reproductive Performance, Tissue Fatty Acid Profile and Desaturase and Elongase mRNAs Expression in Female Zebrafish *Danio rerio*. Aquaculture.

