# Supplementary material for: Dietary Spirulina (Arthrospira platensis) Modulates Survival, Growth, Reproductive Behavior, and Spawning Performance in Zebrafish, Danio rerio
Source: Animals (Basel). 2025 Dec 29;16(1):98. doi: 10.3390/ani16010098 (PMC12784750; doi:10.3390/ani16010098)
Supplement: Supplementary file 1 [file animals-16-00098-s001.zip › animals-3969879-supplementary.pdf]

Dietary spirulina (*Arthrospira platensis*) modulates survival, growth, reproductive behavior, and spawning performance in zebrafish, *Danio rerio*

Ferdinando Flagiello [1], Maria Raggio [1], Marcello Diano [2], Serena Esposito [2], Maddalena Parente [2], Chiara Attanasio [1], Elena De Felice [3], Carla Lucini [1], Stefano Mazzoleni [4], Paolo de Girolamo [1], Livia D'Angelo [1,*] and Antonio Palladino [4,5,*]

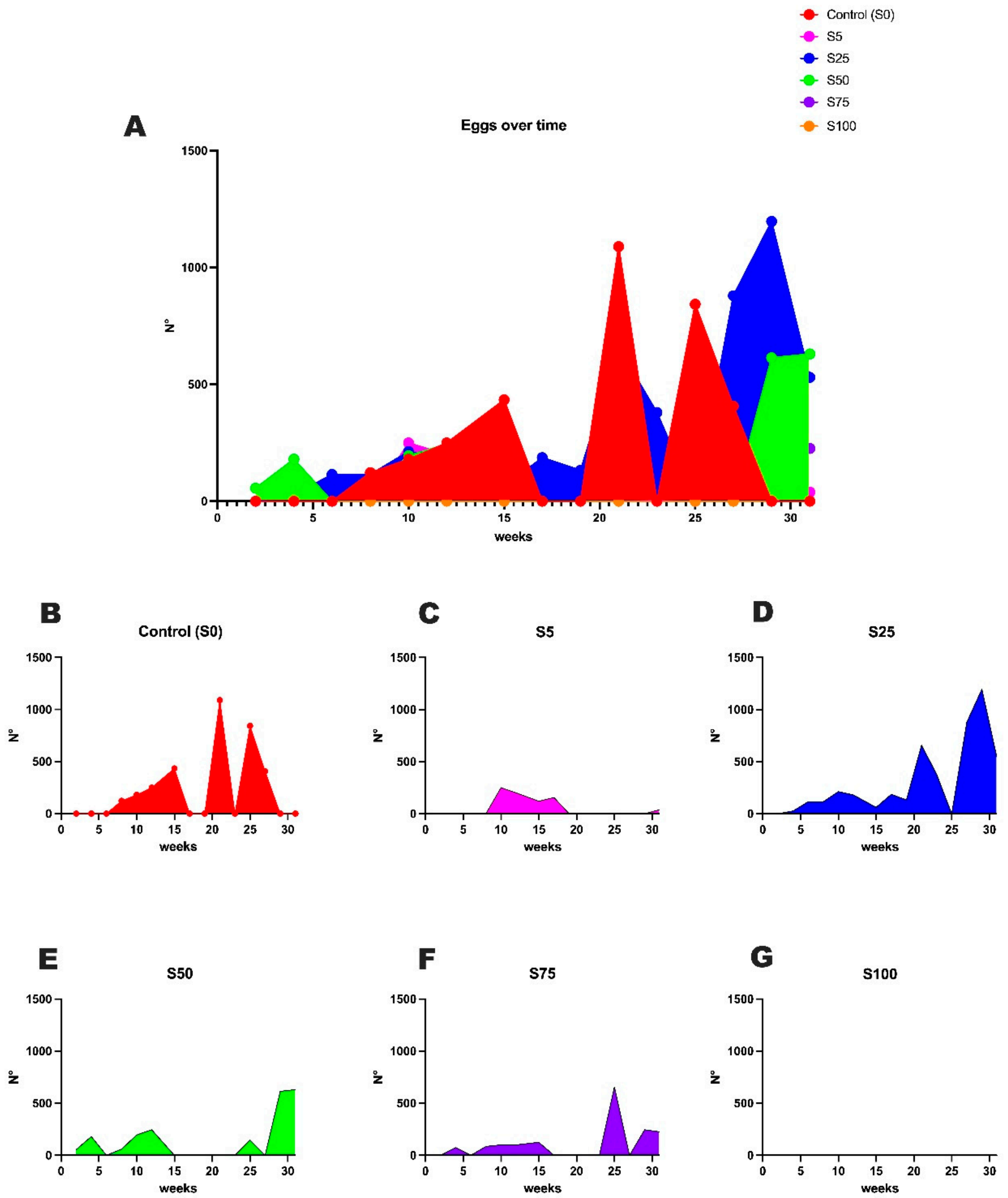

Supplementary Figure S1. Temporal pattern of egg production in the different experimental groups. **A.** Overall distribution of spawned eggs across the experimental period for all dietary treatments. **B.** S0 (control) group: two clear peaks of reproductive activity are evident around weeks 20 and 25, representing the highest spawning intensity during the entire trial. **C.** S5 group: only minimal and irregular spawning events were detected, with a increase before week 10. **D.** S25 group: egg production gradually increased over time, reaching its maximum between weeks 25 and 30. **E.** S50 group: moderate but inconsistent spawning was observed, with a small rise in egg numbers toward the end of the

experimental period. **F.** S75 group: overall egg production remained low, with a peak detected around week 25. **G.** S100 group: this group did not spawn eggs.

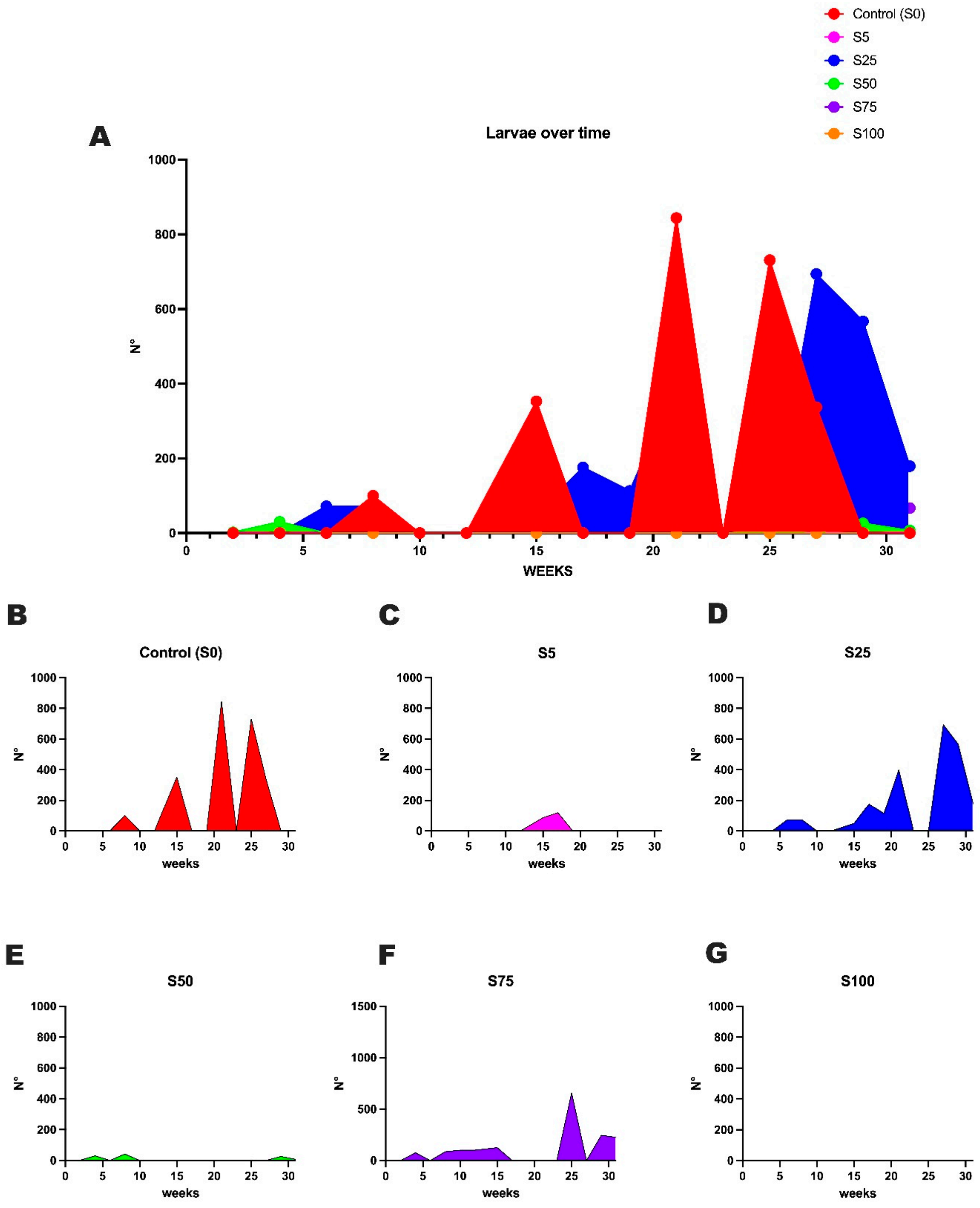

Supplementary Figure S2. Temporal pattern of larval production in the different experimental groups. **A.** Overall distribution of the number of larvae recorded across the experimental period for all dietary treatments. **B.** S0 (control) group: two main peaks are evident around weeks 20 and 25, corresponding to the periods of highest reproductive success, consistent with the egg production trend. **C.** S5 group: the larval output is detected around week 15. **D.**S25 group: a gradual rise in larval numbers is observed toward the end of the trial, with a maximum between weeks 25 and 30 **E.** S50 group: few larvae were recorded throughout the experiment, with slight and irregular increases during early and late weeks. **F.** S75 group: larval output remains generally low, showing only one modest peak around week 25. **G.** S100 group: no larvae detected

Diet	SDS (%)	Spirulina (%)	Proteins (g/100g)	Lipids (g/100g)	Carbohydrates (g/100g)	Fiber (g/100g)	Ashes (g/100g)
Control -S0	100	0	60.00	14.50	11.00	3.00	11.50
S-5	95	5	60.10	14.09	10.95	2.93	11.26
S-25	75	25	60.50	12.45	10.75	2.65	10.32
S-50	50	50	61.00	10.40	10.50	2.30	9.15
S-75	25	75	61.50	8.35	10.25	1.95	7.97
S-100	0	100	62.00	6.30	10.00	1.60	6.80

Supplementary Table S1 : Six experimental diets were formulated by replacing the SDS basal diet with increasing proportions of spirulina powder. The inclusion levels were as follows: Control-S0 (100% SDS), S-5 (95% SDS + 5% spirulina), S-25 (75% SDS + 25% spirulina), S-50 (50% SDS +50% spirulina), S-75 (25% SDS + 75% spirulina), and S-100 (100% spirulina). The proximate composition of each diet was calculated as the weighted mean of the two ingredients according to their inclusion percentage (mass-for-mass substitution). All diets were prepared by homogenizing the ingredients, pelleting and drying at low temperature before storage at 4 °C.
